# Continuous Clarification of Barberry Juice with Pectinase Immobilised by Oxidized Polysaccharides

**DOI:** 10.17113/ftb.59.02.21.6976

**Published:** 2021-06

**Authors:** Seyed Saeid Hosseini, Faramarz Khodaiyan, Seyed Mohammad Mousavi, Seyedeh Zahra Azimi

**Affiliations:** Bioprocessing and Biodetection Laboratory, Department of Food Science and Engineering, University of Tehran, 31587-77871 Karaj, Iran

**Keywords:** barberry juice, pectinase, polyaldehyde derivatives of polysaccharides, immobilisation, packed bed reactor

## Abstract

**Research background:**

Barberry juice is a rich source of bioactive compounds and shows different health properties such as antioxidant and anticancer activities. Clarification, as the removal process of suspended material, is an important step in the production of fruit juice due to its significant effect on the appearance, flavour and commercialisation of juice. Pectinase is the most important enzyme applied in juice clarification that breaks down the pectin polymer structure and reduces the undesirable turbidity. Pectinase immobilisation is a way to overcome free enzyme drawbacks such as instability, high cost, the difficulty of recovery and recyclability. Also, continuous clarification process which is highly preferred in fruit juice industry is not possible without enzyme immobilisation.

**Experimental approach:**

Pectinase enzymes were immobilised on the functionalised glass beads (glass bead with (3-aminopropyl)triethoxysilane) by glutaraldehyde, polyaldehyde derivatives of pullulan and kefiran and the barberry juice was clarified in the batch and continuous processes in a packed bed reactor (PBR). Also, the effect of clarification on the physicochemical and antioxidant properties of the barberry juice samples was evaluated.

**Results and conclusions:**

The optimum conditions for clarification in the PBR were: flow rate 0.5 mL/min, temperature 50 °C and treatment time 63 min. Clarification led to a decrease in turbidity, pH, total soluble solid content, viscosity, total phenolic content and antioxidant activity of the juice samples. Also, this process increased the clarity, acidity, reducing sugar concentration and the lightness parameter of the barberry juice. The greatest effect of clarification on the studied properties of barberry juice was related to the pectinase immobilised by the polyaldehyde of kefiran in the continuous process and both new cross-linkers (polyaldehyde derivatives of pullulan and kefiran) immobilised the enzyme better than the common cross-linker (glutaraldehyde).

**Novelty and scientific contribution:**

For the first time, barberry juice was clarified with pectinase immobilised by polyaldehyde derivatives of pullulan and kefiran and the obtained results showed that the pectinase immobilisation by these new cross-linkers was much more efficient than by the glutaraldehyde as a common cross-linker. These findings can be of use for an industrialised production of fruit juices.

## INTRODUCTION

Barberry (*Berberis vulgaris* L.) fruit, as the largest genus of *Berberidaceae* family, is a rich source of bioactive compounds such as carotenoids, flavonoids, anthocyanins and polyphenols, and therefore shows different health properties, *e.g*. antioxidant and anticancer activities (*1*). Besides, in traditional medicine, this fruit and especially its juice have a wide application in the treatment of colitis, chronic inflammation, high blood pressure, and nervous, liver and heart problems (*2*). Considering these health benefits, it is highly recommended to consume barberry juice as one of the most important nutraceutical beverages. An overview of the production process of barberry juice reveals that its main stages include separation of fruit and washing, raw juice production, clarification, heat treatment and packaging. Among these steps, clarification, which is theoretically defined as the removal of suspended material that causes turbidity and sediment in the product, is particularly important due to its significant effect on the appearance, flavour, customer attention and commercialisation of juice. The main cause of turbidity and sediment is a hydrocolloid named pectin, a polymer of α-1,4-galacturonic acid that makes up approx. 30% of the primary cell wall of plants and enters the product during the juicing process (*3*, *4*); therefore, it is necessary to remove it. In conventional industrial production, this process is done with the help of gelatin, bentonite and different types of membranes (*5*). However, with the advancement of science and the increasing demand for more selective and clean processes in recent years, the enzymes have gained a special place in this process because they have high efficiency, selectivity, low toxicity and also operate under mild reaction conditions (*6*-*8*).

Pectinase is the most important enzyme employed in juice clarification that breaks down the pectin polymer structure and reduces the undesirable turbidity, cloudiness and sediment (*9*). Although the use of free pectinase in the juice production can be considered as a potential method, it has some drawbacks such as instability, high cost and the difficulty of recovery and recyclability, which limit its industrial application (*10*). The way to solve these problems is carrier-bound and carrier-free immobilisation. Carrier-bound immobilisation refers to the process of connecting an enzyme on a suitable support surface by different methods including adsorption, entrapment and covalent binding (*11*-*13*), while carrier-free immobilisation is a direct cross-linking of different enzyme preparations (*14*, *15*). Wahab *et al*. (*16*) stated that the covalent immobilisation of pectinase on the alginate-agar gel by glutaraldehyde increased significantly the stability of the enzymes. Hassan *et al*. (*17*) immobilised pectinase and xylanase enzymes on the alginate beads and reported that the used method was highly efficient in the clarification of apple juice. Benucci *et al*. (*18*) reported that the immobilisation of pectinase and protease enzymes on the chitosan beads by polydialdehyde starch can be considered as an efficient method for clarifying pomegranate juice. There are two key parts of the support and immobilisation method in this definition that determine the capabilities and performance of the system. Regarding support, it would be wise to choose glass beads because the enzymatic system undergoes stress during juice clarification, so it is important to choose a resistant support. In addition to excellent resistance to the stress conditions, the glass beads have received considerable attention as an insoluble support with high capacity to bind enzymes (*19*). Considering the immobilisation method, it seems that covalent binding, as one of the strongest chemical bonds used to immobilise enzymes, is the best method in the industrial applications due to lower probability of the release of enzymes from the support and thus higher stability and reusability (*20*-*22*). For the binding of the enzyme to the support, the existence of a cross-linker is essential. To the best of our knowledge, in all studies where glass bead is used as an enzyme carrier, glutaraldehyde is applied as a cross-linker (*19*, *23*). It is a toxic material in all doses and therefore its use is associated with concerns and challenges. In previous studies, we applied polyaldehyde derivatives of kefiran and pullulan (produced by partial oxidation with sodium periodate) as new cross-linkers to immobilise pectinase on the functionalised glass beads (glass beads with (3-aminopropyl)triethoxysilane) and the obtained results showed that the used polyaldehyde polysaccharides were comparable with glutaraldehyde and also could bind higher amount of enzymes to the support (*24*).

**Fig. 1:**
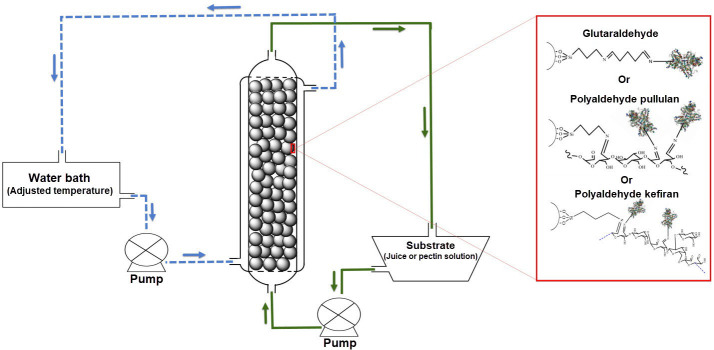
Schematic illustration of the configuration of the continuous packed bed reactor used in the study

One of the most important advantages of immobilised enzymes is the capability to perform a continuous enzymatic process after immobilisation. The first step in performing such continuous enzymatic process is the preparation or manufacture of a bioreactor. Packed bed reactor (PBR) is a simple and inexpensive reactor with a double-walled cylinder packed with support-immobilised enzymes (Fig. 1), which has so far been used successfully in many studies (*11*, *25*). For example, Dal Magro *et al*. (*26*) reported that pectinase immobilised on chitosan particles and used in PBR seems to be a good alternative for large scale application for juice clarification. In another study, Benucci *et al*. (*27*), who used PBR for white wine protein stabilisation by the immobilised cysteine proteases, reported that the enzymatic treatment in PBR can be used as an alternative to bentonite fining.

As mentioned above, the feasibility of using polyaldehyde derivatives of polysaccharides (kefiran and pullulan) as new cross-linkers for the immobilisation of pectinase on the glass beads was investigated in previous studies (*24*, *28*). However, the performance of this immobilisation system under real conditions remains unclear. Without checking the obtained results of juice clarification, the efficiency of this system cannot be judged. Therefore, keeping in mind broad applications of immobilised enzymes in the juice industry, in this study, a continuous clarification of barberry juice using PBR charged with polyaldehyde polysaccharide-immobilised pectinase on glass beads was investigated. Also, the physicochemical and antioxidant properties of the barberry juice samples clarified by polyaldehyde polysaccharide-immobilised pectinase were compared with the samples clarified by free and glutaraldehyde-immobilised enzymes.

## MATERIALS AND METHODS

### Materials

Pectinase enzyme (Pectinex® Ultra Color) with a protein concentration of 80 mg/mL was obtained from Novozymes (Bagsvaerd, Denmark). Also, the preliminary experiments showed that the specific activity of the applied pectinase was (2.93±0.13) U/mg at the temperature 50 °C and pH=5.0 (optimum conditions for free pectinase activity).

Apple pectin with degree of esterification of 72-76% was bought from Herbstreith & Fox KG (Neuenbuerg, Germany). Kefiran and pullulan exopolysaccharides were supplied by Microbiology Laboratory, Department of Food Science and Engineering, University of Tehran (Karaj, Iran) and Herbstreith & Fox KG, respectively. Glass beads with 6 mm diameter were provided by Iran Beads Co. (Esfahan, Iran). The (3-aminopropyl)triethoxysilane (3-APTES), 3,5-dinitrosalicylic acid (DNS), glutaraldehyde, hydroxylamine hydrochloride, sodium periodate and d-(+)-galacturonic acid monohydrate were purchased from Sigma Chemical Co., Merck (St. Louis, MO, USA). All other chemicals and reagents were of analytical grade.

### Preparation of polyaldehyde polysaccharides

The partial oxidation reaction with sodium periodate was applied to produce the polyaldehyde derivatives of kefiran and pullulan (*29*). To produce each polyaldehyde polysaccharide, 5 g sodium periodate powder was dissolved in 100 mL distilled water and the obtained solution was slowly added to 250 mL polysaccharide aqueous solution (4% *m*/*V*) under stirring. After adjusting pH to 5 by adding 1 M sulfuric acid solution, the reaction was continued in the dark for 3 h at (40±2) °C. To precipitate polysaccharides, 96% ethanol in volume ratio *V*(ethanol)/*V*(polysaccharide solution)=2:1 was added and the resulting mixture was stored in a refrigerator (7 °C for 3 days). Afterwards, the oxidised polysaccharides were separated by centrifugation at 10 000×*g* for 20 min, washed three times with distilled water and subsequently freeze-dried for a day. The aldehyde mass fraction in polyaldehyde derivatives of kefiran and pullulan obtained by the method of Kholiya *et al*. (*30*) using hydroxylamine hydrochloride was (23.6±0.9) and (19.9±1.3) %, respectively.

### Immobilisation of pectinase on glass beads

In this study, the cross-linkers of glutaraldehyde, polyaldehyde derivatives of kefiran and pullulan were applied to immobilise pectinase on glass beads according to the method of Gomez *et al*. (*19*). Glass beads (23 cm^3^, approx. 110 beads) were washed with 5% HNO_3_ at approx. 85 °C for 120 min and then dried at 110 °C for 24 h. In the next step, the functionalised glass beads were prepared by mixing them with 10% (*V*/*V*) (3-aminopropyl)triethoxysilane (3-APTES) solution for 3 h at 60 °C and a constant pH=4 (adjusted with 6.0 M HCl). After washing with distilled water and drying at 80 °C for 24 h, the glass beads were slowly stirred in glutaraldehyde solution (2.5% *V*/*V*) or polyaldehyde derivatives of kefiran (2.5% *m*/*V*) or pullulan (2.5% *m*/*V*) for 2.5 h at room temperature (approx. 23 °C). The glass beads were then immersed in pectinase solution diluted with sodium acetate buffer (pH=4 and ratio 1:4 (5 mL enzyme solution with 80 mg/mL protein and 20 mL buffer)) for 5 h at room temperature (approx. 23 °C), then washed with the same buffer and stored at 4 °C.

The protein mass of the glass beads after pectinase immobilisation by glutaraldehyde, polyaldehyde kefiran and polyaldehyde pullulan, respectively, was (4.5±0.7), (57.8±1.1) and (20.1±1.0) mg in 23 cm^3^ glass beads according to Lowry method (*31*) calculated as follows:


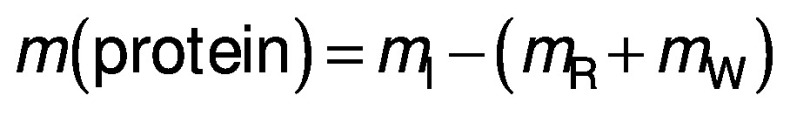


where *m*_I_, *m*_R_ and *m*_W_ are the protein content of the initial, remaining and washing solutions, respectively.

Also, the specific activity of pectinase immobilized by glutaraldehyde, polyaldehyde derivatives of kefiran and pullulan, respectively, was 2.6±0.1 (activity recovery of 87.4%), 1.9±0.2 (activity recovery of 63.8%) and 2.2±0.2 (activity recovery of 75.4%) U/mg protein according to the described method in a previous study (DNS method) (*24*). One unit was defined as the enzyme content required to form 1 μmol/min galacturonic acid under the experimental conditions.

### Kinetic parameters

The Michaelis–Menten constant (*K*_m_) and the maximum reaction rate (*v*_max_) were determined as follows:


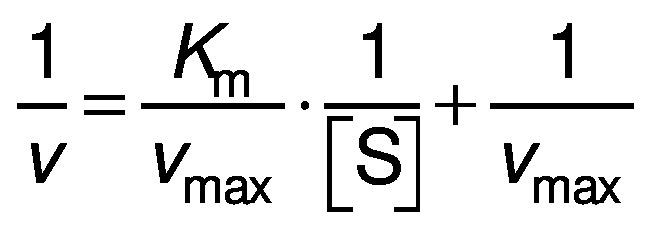


where [S] is pectin concentration (2-20 mg/mL).

It should also be noted that the enzymatic reaction conditions were based on the DNS method (*24*). For this purpose, the enzyme immobilised on 20 cm^3^ glass beads was added to 10 mL of 1% (*m*/*V*) pectin solution prepared with 0.1 M sodium acetate buffer (pH=5.5) and hydrolysed for 15 min at 50 °C. After that, 1 mL of the reaction medium was poured in a test tube and 1 mL of DNS reagent was added to it. The resulting solution was immediately placed in bain-marie (100 °C, 5 min) and then cooled in an ice-water bath. Afterwards, 3 mL distilled water were added to the test tube and its absorbance was determined at 540 nm. d-galacturonic acid (10-1000 µg/mL) was used to draw a standard curve.

### Preparation of barberry juice

The barberry fruits were obtained from a local store in Karaj, Alborz, Iran. The fruits were first separated from the branches and then thoroughly washed. In the next step, barberry juice was made in a household juicer (SJ 3000 W; BRAUN, Shanghai, PR China). The obtained juice was filtered through a Whatman No. 1 paper to separate the insoluble materials and then stored in a refrigerator until next use.

### Packed bed reactor system

To carry out a continuous process, a jacketed glass reactor with an inner diameter of 14 mm and a length of 150 mm was used. This bioreactor was well packed with 23 cm^3^ of pectinase-immobilized glass beads (approx. 110 beads). The packed bed reactor (PBR) was fed from the bottom with 1% (*m*/*V*) buffered pectin solution (pH=3) or barberry juice (pH=3.01), while the temperature was adjusted with the water flow in the outer layer (Fig. 1). The total volume of the PBR was 23.08 cm^3^, while the actual volume of 23 cm^3^ of glass beads (110 beads) was 12.43 cm^3^ and thereby the void volume was 10.65 cm^3^ according to the formula below:


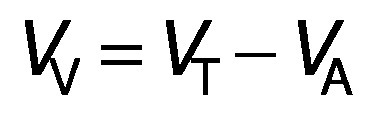


where *V*_V_ and *V*_T_ are the void and total volume of the PBR and *V*_A_ is the actual volume of 23 cm^3^ of glass beads.

### Effect of flow rate, temperature and treatment time on PBR performance

The effect of flow rate, temperature and treatment time on the activity of the immobilized pectinase under a continuous process in the PBR was evaluated using one factor at a time design. For flow rate, this factor was variable (0.1-10 mL/min), while the temperature and pH of the 1% (*m*/*V*) pectin solution were constant at 50 °C and 3.0 (pH of the tested juice), respectively. For each flow rate, the residence time (min) can also be calculated as follows:


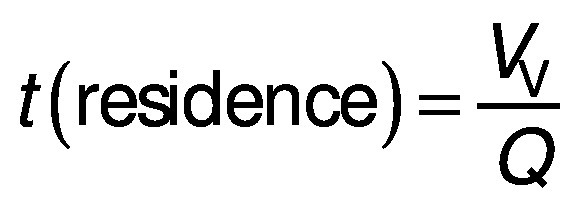


where *V*_V_ is the void volume of the reactor (mL) and *Q* is the flow rate (mL/min).

To determine the best temperature, this factor was varied from 30 to 90 °C, and the flow rate and pH of the 1% (*m*/*V*) pectin solution were considered constant at 0.5 mL/min and 3.0, respectively. To determine the flow rate and temperature, the substrate was pectin solution (1% *m*/*V*) and the response was reducing sugar concentration (mg/mL) determined by the DNS method as stated earlier (*32*).

To determine the best treatment time, this factor was varied from 0 to 105 min, while the flow rate (0.5 mL/min) and temperature (50 °C) were fixed. In this experiment the barberry juice was the substrate and the turbidity was the response. For this purpose, after the clarification at different treatment times, the turbidity of the obtained juice was determined by a portable turbidimeter (350 IR; WTW, Weilheim, Germany) and the results were reported as nephelometric turbidity unit (NTU).

### Volumetric productivity of free and immobilised pectinases in batch and continuous systems

Under the batch conditions, the volumetric productivity of free pectinase and the enzymes immobilised by different cross linkers was determined by adding 56 µL free enzyme or 23 cm^3^ glass bead-immobilised enzymes to 10.65 mL pectin solution and performing the hydrolysis reaction under optimal conditions as mentioned earlier. The volumetric productivity was reported based on mg of the produced reducing sugar per mL of medium per min (mg/(mL·min)). In the continuous process, this parameter was also calculated as follows (*33*):


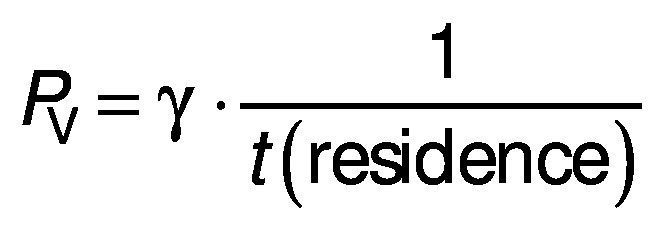


where *γ* is the concentration of the produced reducing sugar (in g/mL).

### Enzymatic clarification of barberry juice

For this purpose, the use of free and immobilised pectinase was investigated under batch and continuous conditions. In the batch process, 56 µL free enzyme or 23 cm^3^ enzyme-immobilised glass beads were immersed into 10.65 mL barberry juice and clarification was done at 50 °C for 63 min. In the case of the continuous operation, the fruit juice was pumped into the PBR with a flow rate of 0.5 mL/min under the same conditions (50 °C, 63 min). Generally, in the current study, seven clarification treatments were considered and the obtained results were compared with untreated juice. The treatments were: raw barberry juice, barberry juice clarified with free pectinase, barberry juice clarified with pectinase immobilized on the glass beads by glutaraldehyde in the batch operation, barberry juice clarified with pectinase immobilised on the glass beads by glutaraldehyde in the continuous operation, barberry juice clarified with pectinase immobilised on the glass beads by polyaldehyde derivative of pullulan in the batch operation, barberry juice clarified with pectinase immobilised on the glass beads by polyaldehyde derivative of pullulan in the continuous operation, barberry juice clarified with pectinase immobilised on the glass beads by polyaldehyde derivative of kefiran in the batch operation, and barberry juice clarified with pectinase immobilised on the glass beads by polyaldehyde derivative of kefiran in the continuous operation.

### Physicochemical characterisation of barberry juice

In this section, the physicochemical properties of raw and treated barberry juice were determined. The turbidity of the juice samples was determined with a portable turbidimeter (350 IR; WTW) and expressed in NTU. The clarity of juice samples diluted with distilled water (fivefold) was obtained by recording the absorbance at 660 nm using a spectrophotometer (CE 2502; CECIL, Cambridge, UK) and calculating the transmittance *T* (in %) according to the equation below:


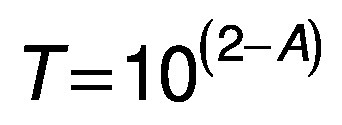


The pH was measured using a pH meter (GLP 22; Crison Instruments, Barcelona, Spain). Total titratable acidity (TTA) was determined by the titration with 0.1 M NaOH in the presence of phenolphthalein as indicator and was expressed in g malic acid per 100 mL juice.

Total soluble solid content (TSS) was determined with a refractometer (Bellingham-Stanley™ Abbe, London, UK) at room temperature and reported in °Brix. The reducing sugar mass concentration (mg/mL) of the samples was measured using the DNS method as mentioned earlier.

The viscosity of the barberry juice samples was determined with a programmable viscometer (DV3TLVTJ0; Ametek Brookfield, Middleborough, MA, USA) at a shear rate of 50 rpm and 23 °C.

The presence of pectin was determined by adding ethanol to juice samples at volume ratio 2:1 and then storing the obtained mixtures at 4 °C for 24 h. In this test, the lack of supernatant formation shows the complete elimination of pectin.

The colour parameters of different samples were obtained by a colour reader (CR-400; CHROMA METER, Tokyo, Japan).

Total phenolic content (TPC) was determined using the Folin-Ciocalteu method (*34*, *35*). A volume of 100 µL of barberry juice was added to 500 µL of 10% Folin-Ciocalteu reagent solution and then 400 µL of 10% (*m*/*V*) sodium carbonate solution were poured into it. After 1 h in darkness, the absorbance was read at 517 nm using a spectrophotometer (CE 2502; CECIL) and the TPC was calculated by the comparison of the determined absorbance with the standard curve obtained for gallic acid (10-100 µg/mL). The TPC was reported as mg gallic acid equivalent per mL barberry juice.

### Determination of antioxidant activity of barberry juice

Antioxidant activity was evaluated by the DPPH radical scavenging activity of barberry juice (*34*, *36*). For this purpose, 100 µL barberry juice diluted with distilled water (200-fold) were added to 1.9 mL of DPPH ethanolic solution (0.1 mM) and the obtained mixture was vortexed. The absorbance of samples was recorded at 517 nm after storing in darkness for 30 min and the DPPH radical scavenging activity (DPPH-RSA) was calculated as follows:


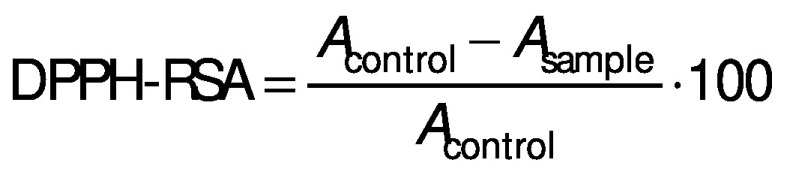


ABTS radical scavenging activity (ABTS-RSA) was determined in a similar way to DPPH-RSA. It should only be noted that to produce ABTS radical solution, 7.4 mM ABTS solution was mixed with 2.6 mM potassium persulfate and after storing in darkness for 18 h, its absorbance was adjusted to 0.70±0.02 at 734 nm by adding distilled water (*34*). Also, in this experiment, the absorbance of the obtained samples was measured at 734 nm.

### Statistical analysis

All the experiments were performed in triplicate and their results were expressed as mean value±S.D. The obtained data were subjected to one-way analysis of variance (ANOVA) using SPSS software v. 20 (*37*). It must also be stated that a significance level of α=0.05 was applied.

## RESULTS AND DISCUSSION

### Kinetic parameters of immobilised pectinases

The kinetic parameters of the immobilised pectinases including Michaelis–Menten constant (*K*_m_) and the maximum reaction rate (*v*_max_) were calculated at temperature of 50 °C and pH=5.5. The obtained results showed *K*_m_ for pectinase immobilised by glutaraldehyde, polyaldehyde derivatives of pullulan and kefiran were (10.1±0.4), (11.2±0.2) and (11.6±0.3) mg/mL, respectively. Also, *v*_max_ value, on protein basis, for pectinase immobilised by the mentioned cross-linkers was (2.9±0.3), (2.2±0.2) and (2.2±0.1) µmol/(min·mg), respectively. The *K*_m_ and *v*_max_ of the pectinase immobilised by polyaldehyde derivatives of pullulan and kefiran were higher and lower than of the pectinase immobilised by glutaraldehyde, which can be due to the diffusion limitation effects created by the polymeric network and three-dimensional structure of the polyaldehyde derivatives of pullulan and kefiran (*28*).

### Effect of flow rate, temperature and treatment time on the activity of immobilised pectinase in PBR

**Fig. 2:**
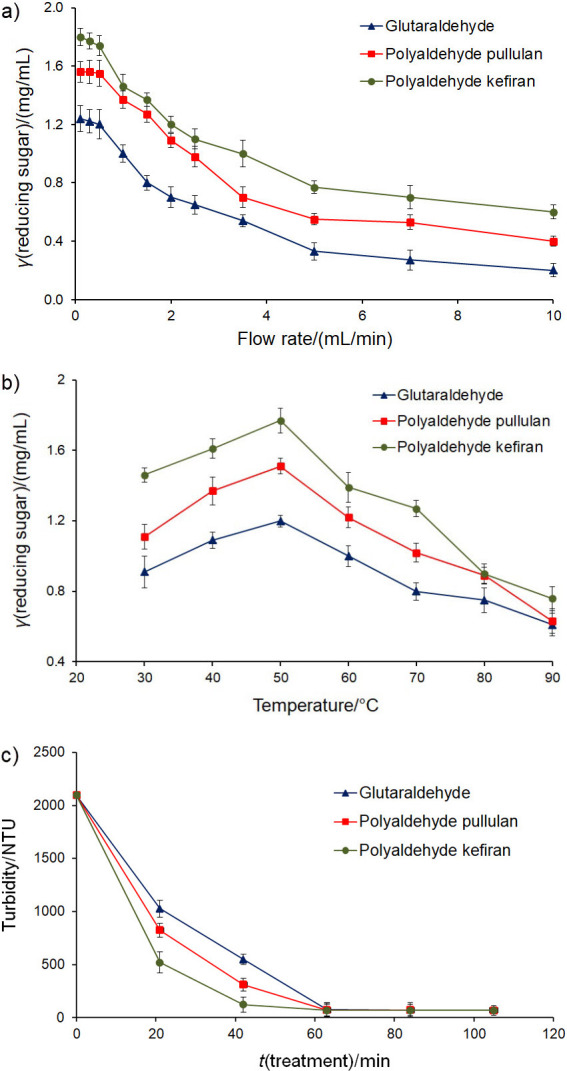
The effect on the packed bed reactor performance of: a) flow rate at 50 °C, b) temperature at flow rate of 0.5 mL/min, and c) treatment time at 50 °C and flow rate of 0.5 mL/min. Glutaraldehyde, polyaldehyde pullulan and polyaldehyde kefiran=the bioreactor packed with pectinase immobilised on glass beads by glutaraldehyde, polyaldehyde derivatives of pullulan and kefiran, respectively. Data are presented as mean value±S.D., *N*=3. NTU=nephelometric turbidity unit*

To determine the effect of flow rate on the activity of immobilised pectinase in the PBR, the reducing sugar production by pectinase immobilised using different cross-linkers was studied (substrate was 1% (*m*/*V*) buffered pectin solution at pH=3 and constant temperature at 50 °C). Fig. 2a shows that the reducing sugar production in a bioreactor packed with pectinase immobilised by polyaldehyde derivative of kefiran was higher than that of the enzymes immobilised by two other cross-linkers (glutaraldehyde and polyaldehyde derivative of pullulan) at all flow rates. This result is probably due to higher protein immobilisation on 23 cm^3^ glass beads by polyaldehyde derivative of kefiran ()57.8±1.1) mg) than polyaldehyde derivative of pullulan ((20.1±1.0) mg) and glutaraldehyde ((4.5±0.7) mg). Also, the reducing sugar production decreased when the flow rate was increased from 0.1 (residence time of 106.5 min) to 10 (residence time of 1.065 min) mL/min, which can be related to shorter contact between the immobilised enzymes and substrate when the flow rate is increased (*38*). However, there is no significant difference between the reducing sugar productions at flow rates from 0.1 to 0.5 mL/min. Therefore, to save time and cost, other experiments in this study were performed at a flow rate of 0.5 mL/min (residence time of 21 min).

Fig. 2b shows the effect of treatment temperature (30-90 °C) on the reducing sugar production by pectinase immobilised using different cross-linkers, while other parameters remained constant (pH=3 and flow rate 0.5 mL/min). In this experiment, similar to the flow rate, the reducing sugar production in the bioreactor packed with pectinase immobilised by polyaldehyde derivative of kefiran was higher than that of the enzyme immobilised by glutaraldehyde and/or polyaldehyde derivative of pullulan at all temperatures. The obtained results indicated that the maximum reducing sugar production was achieved at 50 °C with pectinase immobilised by all three cross-linkers. These results were probably attributed to the optimum temperature for the activity of immobilised pectinase by the mentioned cross-linkers, as shown in a previous study (*24*). However, it seems that the type of immobilisation and the used cross-linker are the key factors in this analysis. For instance, Dal Magro *et al*. (*39*) reported that the optimum temperature for the activity of pectinase immobilised on the glutaraldehyde-activated magnetite was 60 °C, while de Oliveira *et al*. (*11*) stated that this parameter for pectinase immobilised on the alginate beads was 50 °C.

To evaluate the effect of treatment time on the fruit juice clarification in the bioreactor packed with pectinase immobilised on glass beads, the turbidity of the barberry juice was measured at different treatment times (0-105 min), while the flow rate and temperature were constant, 0.5 mL/min and 50 °C, respectively. As observable in Fig. 2c, the turbidity of the samples reached a minimum after 63 min and a further increase in time had no significant effect on this factor. However, the slope of the turbidity reduction in the bioreactor packed with pectinase immobilised by polyaldehyde derivative of kefiran was greater than the other two used cross-linkers, which was possibly due to the higher amount of the enzyme immobilised by this cross-linker. Therefore, all tests on the treated barberry juice were performed after clarification at a flow rate of 0.5 mL/min, temperature of 50 °C and treatment time of 63 min for continuous and the same temperature and treatment time for the batch operation.

### Results of volumetric productivity of free and immobilised pectinase in batch and continuous operations

**Fig. 3:**
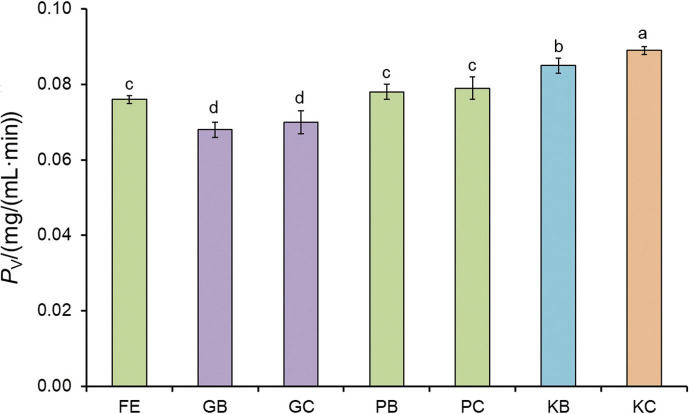
Volumetric productivity of free and immobilised pectinase in the batch and continuous operations. FE=free pectinase, GB=pectinase immobilised by glutaraldehyde in the batch operation, GC=pectinase immobilised by glutaraldehyde in the continuous operation, PB=pectinase immobilised by polyaldehyde derivative of pullulan in the batch operation, PC=pectinase immobilised by polyaldehyde derivative of pullulan in the continuous operation, KB=pectinase immobilised by polyaldehyde derivative of kefiran in the batch operation, KC=pectinase immobilised by polyaldehyde derivative of kefiran in the continuous operation. Data are presented as mean value±S.D., *N*=3. Different letters indicate significant differences (p<0.05)

In this experiment, the volumetric productivity of free pectinase and pectinase immobilised by different cross-linkers in batch and continuous operations was determined and the obtained results are shown in Fig. 3. As can be seen, the maximum volumetric productivity was obtained with pectinase immobilised by polyaldehyde derivative of kefiran in continuous operation, followed by the enzyme immobilised by polyaldehyde derivative of kefiran in batch operation. The higher volumetric productivity of pectinase immobilised by polyaldehyde derivative of kefiran than the other applied cross-linkers was due to the better capacity of this cross-linker for enzyme immobilisation, as mentioned before. Also, the higher volumetric productivity in continuous operation than the batch type was possibly due to better contact between the immobilised pectinase and the substrate in the continuous process. The volumetric productivity of pectinase immobilised by polyaldehyde derivative of pullulan was lower than of pectinase immobilised by polyaldehyde derivative of kefiran, but higher than of glutaraldehyde-immobilised enzyme, due to the amount of the immobilised enzyme. As mentioned earlier, the protein content in free pectinase and in glutaraldehyde-immobilised enzyme was equal and according to Fig. 3, the volumetric productivity of free enzymes was higher than of glutaraldehyde-immobilised ones, which was possibly related to the decrease in enzyme activity due to immobilisation, as shown in previous studies (*20*, *24*).

### Effect of clarification on barberry juice properties

After clarification under optimum conditions (flow rate 0.5 mL/min, temperature 50 °C and treatment time 63 min for continuous and the same temperature and treatment time for batch operation) using free pectinase and pectinase immobilised by different cross-linkers in the batch and continuous operations, the physicochemical, antioxidant and colour properties of the treated barberry juice were evaluated and the obtained results were compared with the untreated sample.

#### Physicochemical and colour properties

Table 1 shows the physicochemical properties of the untreated and clarified juice samples. The most important goal in the fruit juice clarification is to reduce the turbidity and increase clarity. The main cause of this turbidity is the pectin present in the fruit juice and therefore its hydrolysis is necessary (*40*). Table 1 indicates that after clarification, the turbidity of barberry juice samples significantly decreased from 2068.8 to 69.3 NTU and thereby the clarity increased from 28.38 to 73.63%. Also, the obtained results in Table 1 indicate that the applied immobilisation method (the use of polyaldehyde derivatives of kefiran and pullulan as cross-linkers) for the clarification of barberry juice was much more efficient than the common method (the use of glutaraldehyde as cross-linker). Besides, the mentioned results were in accordance with the reported data for the clarification of different fruit juice samples by Bilal *et al*. (*41*) and the clarification of apple juice by Deng *et al*. (*42*).

**Table 1:** Physicochemical characterisation of barberry juice including turbidity, clarity, pH, total titratable acidity (TTA), total soluble solids (TSS), reducing sugar content, viscosity, pectin presence and total phenolic content

Parameter	RBJ	BFP	BGB	BGC	BPB	BPC	BKB	BKC
Turbidity/NTU	(2068.8±0.0)^a^	(72.6±0.4)^d^	(75.1±0.8)^b^	(73.8±0.3)^c^	(73.0±0.4)^d^	(72.7±0.3)^d^	(71.7±0.4)^e^	(69.3±0.6)^f^
Clarity/%	(28.4±0.2)^h^	(61.7±0.2)^e^	(54.1±0.1)^g^	(60.3±0.1)^f^	(63.0±0.1)^d^	(65.13±0.09)^c^	(68.2±0.1)^b^	(73.6±0.1)^a^
pH	(3.21±0.02)^a^	(2.99±0.02)^e^	(3.18±0.01)^b^	(3.14±0.02)^c^	(3.09±0.02)^d^	(2.98±0.04)^e^	(2.77±0.01)^f^	(2.70±0.03)^g^
TTA/(g/100 mL)	(0.75±0.03)^g^	(1.04±0.02)^d^	(0.91±0.03)^f^	(0.97±0.01)^e^	(1.13±0.01)^c^	(1.14±0.02)^c^	(1.21±0.02)^b^	(1.29±0.03)^a^
TSS/ºBrix	(30.5±0.0)^a^	(25.5±0.0)^d^	(29.0±0.0)^b^	(29.0±0.0)^b^	(26.0±0.0)^c^	(25.5±0.0)^d^	(21.5±0.0)^e^	(21.0±0.0)^f^
*γ*(reducing sugar)/(mg/mL)	(53.2±0.2)^h^	(63.4±0.2)^e^	(60.3±0.2)^g^	(62.8±0.1)^f^	(64.1±0.1)^d^	(66.2±0.1)^c^	(69.1±0.1)^b^	(69.57±0.09)^a^
*η*/(mPa·s)	(4.6±0.2)^a^	(4.07±0.04)^b^	(4.2±0.2)^b^	(4.2±0.1)^b^	(4.0±0.1)^bc^	(3.91±0.07)^c^	(3.6±0.1)^d^	(3.3±0.1)^e^
Pectin presence	Yes	No	No	No	No	No	No	No
*γ*(total phenolics)/(mg/mL)	(20.3±0.1)^a^	(19.3±0.1)^b^	(19.6±0.2)^b^	(19.5±0.1)^b^	(19.4±0.1)^b^	(18.9±0.1)^c^	(18.8±0.2)^c^	(18.54±0.04)^d^

RBJ=raw barberry juice, BFP=barberry juice treated with free pectinase, BGB=barberry juice treated with pectinase immobilised by glutaraldehyde in batch operation, BGC=barberry juice treated with pectinase immobilised by glutaraldehyde in continuous operation, BPB=barberry juice treated with pectinase immobilised by polyaldehyde derivative of pullulan in batch operation, BPC=barberry juice treated with pectinase immobilised by polyaldehyde derivative of pullulan in continuous operation, BKB=barberry juice treated with pectinase immobilised by polyaldehyde derivative of kefiran in batch operation, BKC=barberry juice treated with pectinase immobilised by polyaldehyde derivative of kefiran in continuous operation. NTU=nephelometric turbidity unit. Data are presented as mean value±S.D., *N*=3. Different letters in the same row indicate significant differences (p<0.05)

Table 1 shows that the pH of barberry juice samples decreased after clarification, which was in line with the obtained data for the apple juice clarified by the pectinase immobilised in calcium alginate microspheres (*42*). Also, the TTA of the juice samples was significantly increased by clarification, which can be related to the liberation of organic acids, especially galacturonic acid, during pectin hydrolysis by pectinase (*43*).

The obtained results of TSS determination indicate that this parameter decreased after clarification, probably due to the deposition of the suspended solid compounds after pectin hydrolysis and the destruction of the network formed by it. Also, the findings show that clarification led to an increase in the reducing sugar concentration in the treated fruit juice, possibly due to the release of these sugars by pectin hydrolysis.

Pectin has a high water-holding capacity and creates a cohesive network structure. Therefore, pectin hydrolysis by free and/or immobilised pectinase can reduce water holding capacity, release the water into the fruit juice and thereby reduce the viscosity (*11*). As observable in Table 1, the viscosity of the fruit juice samples clarified by all treatments was significantly lower than the untreated sample. Other researchers also reported similar results (*25*, *44*). The results of pectin determination show that no pectin was observed in the fruit juice samples after clarification by free or immobilised pectinase in the batch and continuous operations (Table 1), indicating that pectin was completely hydrolysed.

Three colour parameters including *L* (lightness), *a* (green-red value) and *b* (blue-yellow value) of the untreated barberry juice and the samples clarified by free and immobilised enzymes were evaluated and the obtained data are shown in Table 2. The lightness factor was increased by the enzymatic clarification. This result was expected because a decrease in turbidity and increase in clarity led to the increase in *L* value, which was in agreement with the reported data by de Oliveira *et al*. (*11*) and Sin *et al*. (*44*) for clarified apple and sapodilla juice, respectively. Also, the obtained results indicated that clarification led to a decrease in the green-red value and an increase in the blue-yellow value (Table 2). The decrease in red colour after pectin hydrolysis can be due to the deposition of some antioxidant compounds that are red in the acidic fruit juice.

**Table 2:** The colour parameters of the untreated and treated barberry juice

Parameter	RBJ	BFP	BGB	BGC	BPB	BPC	BKB	BKC
*L*	(10.21±0.03)^e^	(18.1±0.2)^c^	(17.1±0.2)^d^	(17.2±0.1)^d^	(17.5±0.2)^d^	(18.32±0.09)^c^	(20.4±0.2)^b^	(21.1±0.1)^a^
*a*	(22.2±0.1)^a^	(19.2±0.1)^c^	(20.3±0.1)^b^	(20.2±0.1)^b^	(18.4±0.1)^d^	(18.3±0.2)^d^	(17.2±0.1)^e^	(17.14±0.07)^e^
*b*	(4.1±0.1)^c^	(5.0±0.2)^b^	(5.0±0.2)^b^	(5.1±0.1)^b^	(5.1±0.1)^b^	(5.2±0.1)^b^	(5.51±0.08)^a^	(5.7±0.1)^a^

RBJ=raw barberry juice, BFP=barberry juice treated with free pectinase, BGB=barberry juice treated with pectinase immobilised by glutaraldehyde in batch operation, BGC=barberry juice treated with pectinase immobilised by glutaraldehyde in continuous operation, BPB=barberry juice treated with pectinase immobilised by polyaldehyde derivative of pullulan in batch operation, BPC=barberry juice treated with pectinase immobilised by polyaldehyde derivative of pullulan in continuous operation, BKB=barberry juice treated with pectinase immobilised by polyaldehyde derivative of kefiran in batch operation, BKC=barberry juice treated with pectinase immobilised by polyaldehyde derivative of kefiran in continuous operation. Data are presented as mean value±S.D., *N*=3. Different letters in the same row indicate significant differences (p<0.05)

Table 1 shows that TPC was significantly reduced by the clarification, which was in agreement with the reported data by Landbo *et al*. (*43*) and Diano *et al*. (*25*) for the clarified black currant and apple juice, respectively. This was attributed to the deposition of phenolic compounds after pectin hydrolysis and also to the change of the phenolic compound profile during secondary enzymatic activity (*11*).

From the results in Table 1, it is clear that the bioreactor packed with pectinase immobilised by polyaldehyde derivative of kefiran had the greatest effect on the measured parameters, which could be attributed to the more effective enzyme immobilisation by the mentioned cross-linker. Besides, the continuous operation performed better than the batch type, probably due to better contact between the immobilised pectinase and the substrate in the continuous process.

#### Antioxidant properties

**Fig. 4:**
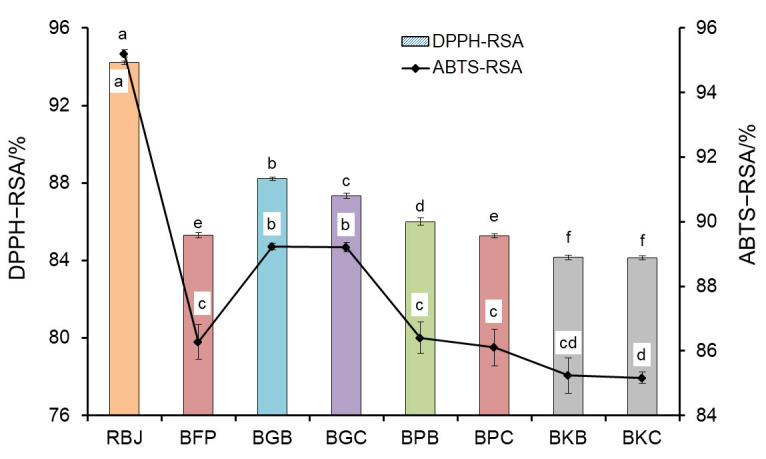
DPPH radical scavenging activity (DPPH-RSA) and ABTS radical scavenging activity (ABTS-RSA) of the untreated and treated barberry juice. RBJ=raw barberry juice, BFP=barberry juice treated with free pectinase, BGB=barberry juice treated with pectinase immobilised by glutaraldehyde in batch operation, BGC=barberry juice treated with pectinase immobilised by glutaraldehyde in continuous operation, BPB=barberry juice treated with pectinase immobilised by polyaldehyde derivative of pullulan in batch operation, BPC=barberry juice treated with pectinase immobilised by polyaldehyde derivative of pullulan in continuous operation, BKB=barberry juice treated with pectinase immobilised by polyaldehyde derivative of kefiran in batch operation, BKC=barberry juice treated with pectinase immobilised by polyaldehyde derivative of kefiran in continuous operation. Data are presented as mean value±S.D., *N*=3. Different letters indicate significant differences (p<0.05)

In this experiment, DPPH and ABTS radical scavenging activities of the untreated and treated barberry juice samples were determined to evaluate their antioxidant properties, and the obtained results are shown in Fig. 4. As can be seen, both parameters were significantly reduced after the clarification. These observations were probably related to the decrease in TPC, which has a direct effect on antioxidant properties (*11*, *45*) and to the deposition of some other antioxidant compounds after pectin hydrolysis. Fig. 4 shows that pectinase immobilised by polyaldehyde derivative of kefiran led to the greatest reduction in antioxidant properties as a result of better ability of the cross-linker to immobilise the enzymes.

## CONCLUSIONS

In this study, different cross-linkers (glutaraldehyde and polyaldehyde derivatives of pullulan and kefiran) were applied to immobilise pectinase on the functionalised glass beads and the barberry juice was clarified in batch and continuous operations using immobilised enzyme. The results show that the best packed bed reactor performance was obtained at a flow rate 0.5 mL/min, temperature 50 °C and treatment time 63 min. Also, the physicochemical, colour and antioxidant properties of the barberry juice significantly changed after clarification. Under the optimum conditions, the turbidity of the fruit juice samples clarified by pectinase immobilised by polyaldehyde derivatives of pullulan and kefiran was significantly lower than of the samples clarified by glutaraldehyde-immobilised pectinase, which was related to the better ability of these new cross-linkers to immobilise the pectinase.

## References

[1] Alizadeh HR Mortezapour H Akhavan HR Balvardi M. Performance of a liquid desiccant-assisted solar juice concentration system for barberry juice. Sol Energy. 2019;184:1–10. 10.1016/j.solener.2019.03.085

[2] Sarraf M Beig Babaei A Naji‐Tabasi S. Investigating functional properties of barberry species: An overview. J Sci Food Agric. 2019;99(12):5255–69. 10.1002/jsfa.9804 31077383

[3] Kazemi M Khodaiyan F Labbafi M Hosseini SS. Ultrasonic and heating extraction of pistachio by-product pectin: Physicochemical, structural characterization and functional measurement. J Food Meas Charact. 2020;14:679–93. 10.1007/s11694-019-00315-0

[4] Asgari K Labbafi M Khodaiyan F Kazemi M Hosseini SS. High-methylated pectin from walnut processing wastes as a potential resource: Ultrasound assisted extraction and physicochemical, structural and functional analysis. Int J Biol Macromol. 2020;152:1274–82. 10.1016/j.ijbiomac.2019.10.224 31751688

[5] Shahrestani H Taheri-Kafrani A Soozanipour A Tavakoli O. Enzymatic clarification of fruit juices using xylanase immobilized on 1,3,5-triazine-functionalized silica-encapsulated magnetic nanoparticles. Biochem Eng J. 2016;109:51–8. 10.1016/j.bej.2015.12.013

[6] Goh WJ Makam VS Hu J Kang L Zheng M Yoong SL Iron oxide filled magnetic carbon nanotube-enzyme conjugates for recycling of amyloglucosidase: Toward useful applications in biofuel production process. Langmuir. 2012;28(49):16864–73. 10.1021/la303046m 23148719

[7] Dal Magro L de Moura KS Backes BE de Menezes EW Benvenutti EV Nicolodi S Immobilization of pectinase on chitosan-magnetic particles: Influence of particle preparation protocol on enzyme properties for fruit juice clarification. Biotechnol Rep (Amst). 2019;24:e00373. 10.1016/j.btre.2019.e00373 31516853 PMC6728273

[8] Cerreti M Liburdi K Benucci I Esti M. The effect of pectinase and protease treatment on turbidity and on haze active molecules in pomegranate juice. Lebensm Wiss Technol. 2016;73:326–33. 10.1016/j.lwt.2016.06.030

[9] Sojitra UV Nadar SS Rathod VK. A magnetic tri-enzyme nanobiocatalyst for fruit juice clarification. Food Chem. 2016;213:296–305. 10.1016/j.foodchem.2016.06.074 27451184

[10] Azar RISL da Luz Morales M Maitan-Alfenas GP Falkoski DL Alfenas RF Guimaraes VM. Apple juice clarification by a purified polygalacturonase from *Calonectria pteridis.* Food Bioprod Process. 2020;119:238–45. 10.1016/j.fbp.2019.11.013

[11] de Oliveira RL Dias JL da Silva OS Porto TS. Immobilization of pectinase from *Aspergillus aculeatus* in alginate beads and clarification of apple and umbu juices in a packed bed reactor. Food Bioprod Process. 2018;109:9–18. 10.1016/j.fbp.2018.02.005

[12] Rong J Zhou Z Wang Y Han J Li C Zhang W Immobilization of horseradish peroxidase on multi-armed magnetic graphene oxide composite: improvement of loading amount and catalytic activity. Food Technol Biotechnol. 2019;57(2):260–71. 10.17113/ftb.57.02.19.5832 31537975 PMC6718962

[13] Cui J Jia S. Organic–inorganic hybrid nanoflowers: A novel host platform for immobilizing biomolecules. Coord Chem Rev. 2017;352:249–63. 10.1016/j.ccr.2017.09.008

[14] Cui JD Jia SR. Optimization protocols and improved strategies of cross-linked enzyme aggregates technology: Current development and future challenges. Crit Rev Biotechnol. 2015;35(1):15–28. 10.3109/07388551.2013.795516 23886350

[15] Bilal M Cui J Iqbal HM. Tailoring enzyme microenvironment: State-of-the-art strategy to fulfill the quest for efficient bio-catalysis. Int J Biol Macromol. 2019;130:186–96. 10.1016/j.ijbiomac.2019.02.141 30817963

[16] Abdel Wahab WA Karam EA Hassan ME Kansoh AL Esawy MA Awad GE. Optimization of pectinase immobilization on grafted alginate-agar gel beads by 2^4^ full factorial CCD and thermodynamic profiling for evaluating of operational covalent immobilization. Int J Biol Macromol. 2018;113:159–70. 10.1016/j.ijbiomac.2018.02.086 29458101

[17] Hassan SS Williams GA Jaiswal AK. Computational modelling approach for the optimization of apple juice clarification using immobilized pectinase and xylanase enzymes. Curr Res Food Sci. 2020;3:243–55. 10.1016/j.crfs.2020.09.003 33251526 PMC7680705

[18] Benucci I Mazzocchi C Lombardelli C Cacciotti I Esti M. Multi-enzymatic systems immobilized on chitosan beads for pomegranate juice treatment in fluidized bed reactor: Effect on haze-active molecules and chromatic properties. Food Bioprocess Technol. 2019;12:1559–72. 10.1007/s11947-019-02315-w

[19] Gomez JL Bodalo A Gomez E Bastida J Hidalgo AM Gomez M. Immobilization of peroxidases on glass beads: an improved alternative for phenol removal. Enzyme Microb Technol. 2006;39(5):1016–22. 10.1016/j.enzmictec.2006.02.008

[20] Lei Z Jiang Q. Synthesis and properties of immobilized pectinase onto the macroporous polyacrylamide microspheres. J Agric Food Chem. 2011;59(6):2592–9. 10.1021/jf103719t 21341670

[21] Dahiya P Chand S Dilbaghi N. Immobilization of organic solvent-tolerant lipase from *Pseudomonas mendocina* M-37 with potential synthetic activities. Food Technol Biotechnol. 2014;52(3):368–75.

[22] Cui J Ren S Sun B Jia S. Optimization protocols and improved strategies for metal-organic frameworks for immobilizing enzymes: Current development and future challenges. Coord Chem Rev. 2018;370:22–41. 10.1016/j.ccr.2018.05.004

[23] Bodalo A Gomez E Gomez JL Bastida J Maximo MF Diaz F. A comparison of different methods of β-galactosidase immobilization. Process Biochem. 1991;26(6):349–53. 10.1016/0032-9592(91)85025-J

[24] Hosseini SS Khodaiyan F Mousavi SME Azimi SZ Gharaghani M. Immobilization of pectinase on the glass bead using polyaldehyde kefiran as a new safe cross-linker and its effect on the activity and kinetic parameters. Food Chem. 2020;309:125777. 10.1016/j.foodchem.2019.125777 31699560

[25] Diano N Grimaldi T Bianco M Rossi S Gabrovska K Yordanova G Apple juice clarification by immobilized pectolytic enzymes in packed or fluidized bed reactors. J Agric Food Chem. 2008;56(23):11471–7. 10.1021/jf8019437 18986151

[26] Dal Magro L Pessoa JP Klein MP Fernandez-Lafuente R Rodrigues RC. Enzymatic clarification of orange juice in continuous bed reactors: Fluidized-bed *versus* packed-bed reactor. Catal Today. 2021;362:184–91. 10.1016/j.cattod.2020.02.003

[27] Benucci I Lombardelli C Liburdi K Acciaro G Zappino M Esti M. Immobilised native plant cysteine proteases: packed-bed reactor for white wine protein stabilisation. J Food Sci Technol. 2016;53(2):1130–9. 10.1007/s13197-015-2125-4 27162393 PMC4837734

[28] Hosseini SS Khodaiyan F Mousavi SM Kennedy JF Azimi SZ. A health-friendly strategy for covalent-bonded immobilization of pectinase on the functionalized glass beads. Food Bioprocess Technol. 2021;14:177–86. 10.1007/s11947-020-02524-8

[29] Guo J Ge L Li X Mu C Li D. Periodate oxidation of xanthan gum and its crosslinking effects on gelatin-based edible films. Food Hydrocoll. 2014;39:243–50. 10.1016/j.foodhyd.2014.01.026

[30] Kholiya F Chaudhary JP Vadodariya N Meena R. Synthesis of bio-based aldehyde from seaweed polysaccharide and its interaction with bovine serum albumin. Carbohydr Polym. 2016;150:278–85. 10.1016/j.carbpol.2016.05.022 27312639

[31] Lowry OH Rosebrough NJ Farr AL Randall RJ. Protein measurement with the Folin phenol reagent. J Biol Chem. 1951;193:265–75. 10.1016/S0021-9258(19)52451-6 14907713

[32] Miller GL. Use of dinitrosalicylic acid reagent for determination of reducing sugar. Anal Chem. 1959;31(3):426–8. 10.1021/ac60147a030

[33] Singh RS Saini GK Kennedy JF. Continuous hydrolysis of pullulan using covalently immobilized pullulanase in a packed bed reactor. Carbohydr Polym. 2011;83(2):672–5. 10.1016/j.carbpol.2010.08.037

[34] Kazemi M Khodaiyan F Hosseini SS Najari Z. An integrated valorization of industrial waste of eggplant: Simultaneous recovery of pectin, phenolics and sequential production of pullulan. Waste Manag. 2019;100:101–11. 10.1016/j.wasman.2019.09.013 31526957

[35] Feizy J Jahani M Ahmadi S. Antioxidant activity and mineral content of watermelon peel. J Food Bioprocess Eng. 2020;3(1):35–40. 10.22059/JFABE.2020.75811

[36] Karimi S Sharifzadeh S Abbasi H. Sequential ultrasound-microwave assisted extraction as a green method to extract essential oil from *Zataria multiflora.* J Food Bioprocess Eng. 2020;3(2):101–9. 10.22059/JFABE.2020.308833.1064

[37] IBM SPSS. v. 20, IBM Corp., Armonk, NY, USA; 2020. Available from: https://www.ibm.com/products/spss-statistics.

[38] Ansari SA Husain Q. Lactose hydrolysis by β galactosidase immobilized on concanavalin A-cellulose in batch and continuous mode. J Mol Catal, B Enzym. 2010;63:68–74. 10.1016/j.molcatb.2009.12.010

[39] Dal Magro L Silveira VCC de Menezes EW Benvenutti EV Nicolodi S Hertz PF Magnetic biocatalysts of pectinase and cellulase: Synthesis and characterization of two preparations for application in grape juice clarification. Int J Biol Macromol. 2018;115:35–44. 10.1016/j.ijbiomac.2018.04.028 29634966

[40] Sharma HP Patel H Sharma S. Enzymatic extraction and clarification of juice from various fruits – A review. Trends Post Harvest Technol. 2014;2(1):1–14.

[41] Bilal M Asgher M Iqbal HMN Hu H Zhang X. Delignification and fruit juice clarification properties of alginate-chitosan-immobilized ligninolytic cocktail. Lebensm Wiss Technol. 2017;80:348–54. 10.1016/j.lwt.2017.02.040

[42] Deng Z Wang F Zhou B Li J Li B Liang H. Immobilization of pectinases into calcium alginate microspheres for fruit juice application. Food Hydrocoll. 2019;89:691–9. 10.1016/j.foodhyd.2018.11.031

[43] Landbo AKR Pinelo M Vikbjerg AF Let MB Meyer AS. Protease-assisted clarification of black currant juice: synergy with other clarifying agents and effects on the phenol content. J Agric Food Chem. 2006;54(18):6554–63. 10.1021/jf060008d 16939309

[44] Sin HN Yusof S Hamid NSA Rahman RA. Optimization of enzymatic clarification of sapodilla juice using response surface methodology. J Food Eng. 2006;73(4):313–9. 10.1016/j.jfoodeng.2005.01.031

[45] Su MS Silva JL. Antioxidant activity, anthocyanins, and phenolics of rabbiteye blueberry (*Vaccinium ashei*) by-products as affected by fermentation. Food Chem. 2006;97(3):447–51. 10.1016/j.foodchem.2005.05.023

